# Conflict (Rohingya, COVID-19, and coup) in Myanmar: unmet need of mental health

**DOI:** 10.1017/gmh.2021.22

**Published:** 2021-07-02

**Authors:** Sheikh Shoib, S.M. Yasir Arafat, Myat Thuzar

**Affiliations:** 1 LNM Hospital Srinagar – Psychiatry, India; 2 Enam Medical College and Hospital Ringgold Standard Institution, Savar 1340, Bangladesh; 3 Mental Health Hospital, Mandalay, Myanmar

Myanmar is a Southeast Asian country with a population of 54 million and a history of the longest-running civil war due to rambling ethnic conflict. The situation has been worsened by issues like migration issues of Rohingyas, the coronavirus disease-2019 (COVID-19) pandemic, and the recent coup (1 February 2021). The conflict has both immediate and enduring effects on mental health. One recent systematic review found that the prevalence of psychiatric disorders [depressive disorder, anxiety disorder, post-traumatic stress disorder (PTSD), bipolar disorder, and schizophrenia] was 22⋅1% among conflict-affected people (Charlson *et al*., 2019). Conflict has a clear negative impact on the mental health of children and adolescents also (Pritchard and Choonara, 2017). A higher prevalence of PTSD was found among the children and adolescents living in areas with armed conflict such as 23%–70% in Palestine, 10%–30% in Iraq, 5%–8% in Israel, and 54%–62% in Rwanda (Pritchard and Choonara, 2017). Mental health is an area in desperate need of attention in Myanmar at least in the current socio-political situation.

There is an instant need for the advance of mental health services in Myanmar complemented by public participation, awareness agendas, and mental health restoration services. Both the government and non-governmental organizations have implemented a community-based psychosocial intervention model for the provision of psychosocial support in the country (Vukovich and Mitchell, 2015). However, it would be challenging to implement and to continue the same intervention model during an ongoing armed conflict. There is also an urgent need for researchers, clinicians, policymakers for devising policies and interventions in the context of the prevailing mental health status of the Myanmar population. Stability and piecing together the civil society are necessary to reduce the burden albeit; it is a further challenge during the current conflict of Myanmar. International humanitarian support is needed for ongoing conflict turmoil and COVID-19 response stirring in Myanmar. An emergency as well as easily accessible team with the ability to support acutely stressed and grieved people could be formulated as per the World Health Organization recommendation (Van Ommeren, 2019). In Myanmar, the global mental health emergency imposed by the ongoing political conflict, and COVID-19 pandemic has met and exacerbated a complex humanitarian crisis, adding to the growing mental health burden in the country. The global health community and international organizations need to pay attention to Myanmar. It is both an issue of human health and human rights, and an essential need for the people in Myanmar.
